# A Practical Approach for Targeting Structural Variants Genome-wide in Plasma Cell-free DNA

**DOI:** 10.21203/rs.3.rs-3492157/v1

**Published:** 2024-01-05

**Authors:** Hisashi Tanaka, Michael Murata, Fumie Igari, Ryan Urbanowicz, Lila Mouakkad, Sungjin Kim, Zijing Chen, Dolores Di Vizio, Edwin Posadas, Armando Giuliano

**Affiliations:** Cedars-Sinai Medical Center; Cedars-Sinai Medical Center; Juntendo University; Cedars-Sinai Medical Center; Cedars-Sinai Medical Center; Cedars-Sinai Medical Center; Cedars-Sinai Medical Center; Cedars-Sinai Medical Center; Cedars-Sinai Medical Center; Cedars-Sinai Medical Center

## Abstract

Interrogating plasma cell-free DNA (cfDNA) to detect cancer offers promise; however, no current tests scan structural variants (SVs) throughout the genome. Here, we report a simple molecular workflow to enrich a tumorigenic SV (DNA palindromes/fold-back inversions) that often demarcates genomic amplification and its feasibility for cancer detection by combining low-throughput next-generation sequencing with automated machine learning (Genome-wide Analysis of Palindrome Formation, GAPF-seq). Tumor DNA signal manifested as skewed chromosomal distributions of high-coverage 1-kb bins (HCBs), differentiating 39 matched breast tumor DNA from normal DNA with an average AUC of 0.9819. In a proof-of-concept liquid biopsy study, cfDNA from 0.5 mL plasma from prostate cancer patients was sufficient for binary classification against matched buffy coat DNA with an average AUC of 0.965. HCBs on the X chromosome emerged as a determinant feature and were associated with AR amplification. GAPF-seq could generate unique cancer-specific SV profiles in an agnostic liquid biopsy setting.

## Introduction

Genome instability is a hallmark of cancer.^1–5^ Changes at the single nucleotide level (i.e., mutations) can activate oncogenes or inactivate tumor suppressor genes. Equally common are structural rearrangements of large chromosomal segments (i.e., structural variants, SVs), such as duplications, deletions, and translocations. These genetic changes are the primary drivers of malignant transformation, disease progression, and therapy resistance. In addition to their roles in cancer biology, tumor-specific DNA changes are targets for detecting cancer, which has increasingly been sought after in the context of liquid biopsy.^6,7^ Liquid biopsy holds promise as less invasive and more repeatable than currently available assays at the clinic (e.g., needle biopsies or imaging scans). Historically, blood has been a source of protein biomarkers such as prostate-specific antigen (PSA).^8^ More recently, with the advent of DNA-analyses technologies for cancer genomes, DNA released into the blood from tumor cells (i.e., circulating tumor DNA, ctDNA) has become a mainstream target for developing liquid biopsy-based clinical tests. Several Food and Drug Administration (FDA) approved tests are currently in clinical use.^9–14^ These tests identify tumor-specific mutations in cell-free DNA (cfDNA) isolated from plasma, either by a single-gene platform or by Next-Generation Sequencing (NGS) for multiple cancer genes (i.e., targeted sequencing). While these DNA tests report residual tumor burden and actionable mutations for advanced cancer patients, mutation detection in plasma cfDNA would also be feasible for cancer screening in the general population. However, even non-malignant hematopoietic cells accumulate clonal mutations in cancer-associated genes such as p53 and *KRAS*,^15–17^ which confound the mutations as effective cancer-specific biomarkers. Furthermore, because ctDNA is only a small fraction of the total cfDNA (tumor fraction, TF) in blood, particularly in patients with early-stage tumors,^18^ ctDNA detection requires very high sensitivity.

Alternatively, SVs are plausible biomarkers for cancer, but have not been pursued for liquid biopsy targets as rigorously as mutations and other emerging targets based on epigenetic features, such as DNA methylation and fragmentation patterns. SVs often manifest as DNA copy number alterations (CNA), but detecting CNA also suffers from very low TF in plasma.^19,20^ Tumor-specific translocations can be detected by discordant mate pairs in whole genome sequencing (WGS) data^21^ However, the detection of SVs by mate-pair analyses requires deep coverage and would be computationally very challenging in cfDNA with low TF. In silico selection of naïve short DNA fragments (<150 bp) for WGS libraries could enhance CNA signal from the tumor fraction.^22^ However, cfDNA fragment sizes could be more variable (long and short) in cancer patients’ plasma than in healthy individuals’ plasma^23,24^. Tumor-derived high molecular weight DNA in plasma has also been documented.^25^

**Figure 1:**
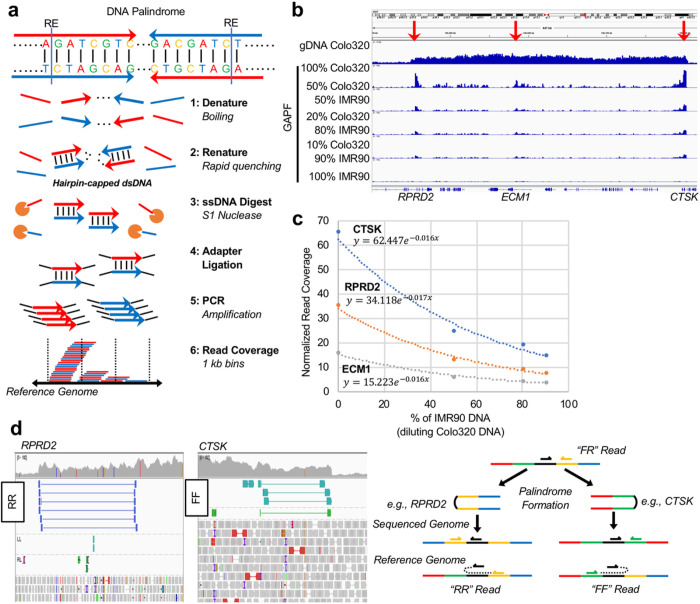
Genome-wide Analysis of Palindrome Formation (GAPF) enriches structural variants. **a** DNA palindromes are genomic regions with sequences that are reverse compliments of each other and could span large genomic segments. Genomic DNA was first digested with restriction enzymes (RE), by which only palindromic junctions folded-back to hairpin-capped dsDNA after denaturing DNA by boiling and rapidly renaturing in an ice water quench. Non-palindromic DNA, including DNA liberated from palindromic junctions by RE, was digested by S1 nuclease. Note that S1 also digested the hairpin part of DNA from palindromic junctions. Following adapter ligation to dsDNA and amplification by PCR was done as a part of NGS library construction. Read coverage was quantified in 1 kb bins genome-wide. **b** Read coverage in 1 kb bins for genomic or GAPF DNA is visualized using the Integrative Genomics Viewer (IGV). Colo320 colorectal adenocarcinoma cancer cell line DNA is diluted by IMR90 normal fibroblast DNA prior to GAPF. Read coverage in GAPF data show peaks at known palindromic junctions on chr1 at RPRD2, ECM1, and CTSK. **c** The GAPF read coverage decreases following an exponential trend at RPRD2 (R2 = 0.9908), ECM1 (R2 = 0.9848), and CTSK (R2 = 0.9776) as the amount of diluting IMR90 normal DNA increases. **d** Paired sequencing reads can have anomalous orientations such as “reverse-reverse” (RR) or “forward-forward” (FF) at sites of structural rearrangements near known palindromic junctions in Colo320 cancer cell line DNA (left). A schematic for how palindromes create RR or FF read pairs is presented (right).

Low TF can be overcome by enriching cancer DNA-specific features in vitro by processing cfDNA. To test the idea, we turned to our SV-targeting technology called Genome-wide Analysis of Palindrome Formation (GAPF). The targeted SVs are DNA palindromes (also called inverted repeats or fold-back inversions),^26^ defined by DNA sequences identical to their reverse complements (Fig. 1A).^27–29^ The procedure for enriching DNA palindromes is relatively simple and can be done in vitro from genomic DNA. While large (>10 kb) germline palindromes are characteristic of sex chromosomes,^30,31^ DNA palindromes arise *de novo* in cancer by the inverted duplication of single-copy DNA sequences.^27^ Multiple palindromes can form during Breakage-Fusion-Bridge (BFB) cycles initiated by a chromosome with two centromeres (i.e. dicentric chromosomes), arising from common processes in genome instability such as illegitimate repair of DNA double-strand breaks (DSBs), fusions of dysfunctional and critically short telomeres, and translocation gaining two centromere-bearing chromosomes.^32,33^ Importantly, anaphase bridges between dividing nuclei, an indication of segregating dicentric chromosomes, were observed in preneoplastic regions,^34^ suggesting that DNA palindromes can arise very early during carcinogenesis, and thus can potentially be an excellent cancer biomarker.

## Results

### Palindrome Detection by GAPF-seq

A protocol for the Genome-wide Analysis of Palindrome Formation (GAPF) was modified to contend with the low amounts of cell-free DNA (cfDNA) in plasma (Fig. 1A). Briefly, input genomic DNA (as low as 30 ng) was first digested with relatively infrequent cutting enzymes, boiled for denaturation, and immediately quenched in ice to facilitate the rapid self-annealing of palindromic sequences (i.e., snap-back).^27,35^ The DNA was then digested using the single-strand specific nuclease S1. The junctions of palindromic DNA can self-anneal following denaturing by boiling to re-form double-stranded DNA (dsDNA) and resistant to S1, whereas non-palindromic sequences will be digested by S1 after denaturation. The remaining dsDNA was ligated with sequencing adaptors and amplified during library construction. Resulting sequencing libraries should thus be enriched with palindromic junctions.

The sequencing reads from GAPF-processed DNA were aligned to the hg38 human reference genome and counted in 1 kb non-overlapping bins (Fig. 1A). An accumulation of reads in these 1 kb bins leads to high coverage in the bin and so, high coverage bins (HCBs) could indicate the presence of DNA palindromes.^29,36^ Indeed, HCBs were seen in known palindromic junctions on chr1 at *RPRD2, ECM1*, and *CTSK* in Colo320DM cell line DNA (Fig. 1B).^29^ To simulate low tumor fraction (TF) as expected from plasma cfDNA, Colo320DM DNA was diluted with IMR90 normal fibroblast DNA *in vitro* prior to GAPF. HCBs were detected at *RPRD2, ECM1*, and *CTSK* for all dilutions. No such peaks in this region were apparent in GAPF-seq data from IMR90 DNA (Fig. 1B, 100% IMR90). The average normalized read coverage (number of sequencing reads per bin divided by a per-million scaling factor) for HCBs at *CTSK* was 65.5 for 100% Colo320 input DNA and decreased exponentially by dilution (Fig. 1C). This exponential trend was similarly observed for *RPRD2* and *ECM1*. The lowest peak was detected at *ECM1* in 10% Colo DNA with an average read coverage of 3.67. Since the average read coverage genome-wide was approximately 0.31, GAPF-seq can still reliably detect palindromic signals after 10-fold dilution.

With paired-end sequencing, the relative orientation of mates can corroborate the presence of palindromes at amplified genomic segments. From WGS data, mates facing in the same direction (e.g., forward-forward, FF, and reverse-reverse, RR) relative to the reference genome can be found at the ends of the amplified region within 1q21. Identifying the FF or RR read pairs was possible in publicly-available deep coverage WGS data (Fig. 1D)^37^, but not our shallow WGS, possibly due to the underrepresentation of secondary structure-forming DNA in sequencing libraries. Navigating the secondary structure of palindromic DNA during PCR, a part of library construction processes, is known to be challenging.^38,39^

### GAPF-seq for Breast Tumor DNA

**Figure 2:**
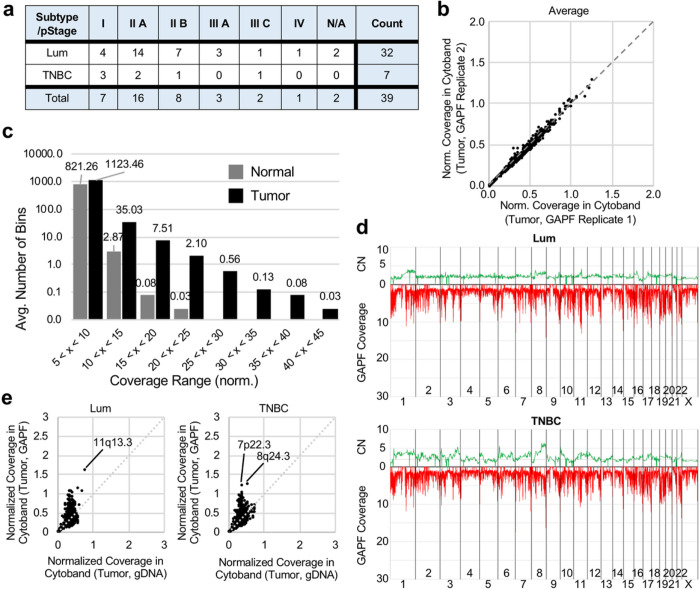
High coverage 1 kb GAPF bins are tumor-specific. **a** A table showing the breast cancer subtype and pathologic stage (pStage) for 39 breast tumors used for GAPF. **b** The GAPF read coverage in cytobands is normalized by the size of the cytoband (in bp) and averaged for the seven (7) duplicates. The normalized read coverage in each cytoband for the first set of replicates is plotted against the duplicates with an intraclass correlation coefficient of 0.9971 (95% CI: 0.9966–0.9974). **c** The average number of 1 kb bins with normalized read coverages in different ranges from 39 breast tumors and matched normal. Out of the 3,209,513 bins in the human genome, the vast majority have very low read coverage in the range between 0 and 5 (not shown). At higher coverage ranges, the average number of bins with high coverage is greater in tumor than normal. **d** The average copy number (CN) is plotted against the average GAPF read coverage for luminal (Lum) breast tumors (top, n = 26) and triple negative breast cancer (TNBC) tumors (bottom, n = 5). **e** The GAPF read coverage in cytobands is normalized by the size of the cytoband (in bp). The normalized read coverage in each cytoband for genomic DNA (gDNA) is plotted against GAPF DNA and averaged for Lum (left, n = 26) or TNBC (right, n = 5) tumors.

To evaluate the ability of GAPF-seq to differentially call tumor DNA and normal DNA, we first collected tumor and normal GAPF-seq data from 39 pairs of fresh frozen breast tumors and matched leukocytes (buffy coat) (Supp Table 1). Breast tumors were either triple-negative breast cancer (TNBC) or luminal breast cancer subtypes, with stages ranging from Stage I to Stage IV (Fig. 2A). After GAPF, sequencing reads were counted in 1 kb bins genome-wide and the read coverage was calculated as described above (Fig. 1A). The average read coverage in each cytoband (instead of 1 kb bins) throughout the genome was evaluated to test the reproducibility of GAPF-seq using replicates from seven pairs of tumor and normal DNA (Fig. 2B, Supp Fig. 1). The read coverage at the cytoband level was almost identical, with an intraclass correlation coefficient of 0.9971 (95% CI: 0.9966–0.9974), confirming the reproducibility of the assay.

A vast majority of 1 kb bins (approximately 3,130,000 bins out of 3,209,513 total bins) from either breast tumor or buffy coat had an average read coverage less than 5, likely due to the digestion of non-palindromic sequences by S1 nuclease. However, the average number of 1 kb bins with read coverage > 5 differed between tumor and normal samples (Fig. 2C). Especially at higher coverage ranges (read coverage > 10), tumor GAPF-seq data exhibited a greater number of bins than normal GAPF-seq data, suggesting the presence and enrichment of tumor-specific palindromes. This observation formed the basis for defining HCBs to differentiate tumor and normal DNA.

Since DNA palindromes are often associated with genomic amplification, the average copy number (CN) of chromosomal segments from shallow WGS data was plotted against the read coverage of GAPF-seq data for 26 luminal or 5 TNBC tumors (Fig. 2D). Overall, TNBC showed more CNA than luminal breast tumors, consistent with previous reports.^40^ Genome-wide GAPF read coverage appeared indistinguishable between subtypes. However, by looking at GAPF-seq and WGS data from individual pairs, we noticed unique subtype coverage patterns. For example, for patient 14–990 with TNBC, there was significant CNA genome-wide (Supp Fig. 2A). Notably, there was enrichment of DNA by GAPF-seq in cytoband 8q24.3 that correlated to increased CN. In contrast, a patient with luminal breast cancer (14–752) presented CNA and increased GAPF coverage that were localized primarily to cytoband 11q13 (Supp Fig. 2B). The coverage from GAPF-seq data was plotted against that from WGS at the cytoband level (Fig. 2E). Overall, cytobands 11q13.2–5 were enriched in luminal tumors, while cytoband 8q24.3 was predominant in TNBC.

### Tumor DNA Calling Algorithm by GAPF-seq

Upon further inspection of regions of high GAPF coverage in the genome, it was found that several bins were commonly enriched in both tumor and normal samples (Supp Fig. 3). We considered the possibility We considered the possibility of germline palindromes.^41,42^ Alternatively, particular DNA sequences were relatively more resistant to denaturation. As a result, these sequences would be resistant to S1 nuclease and subsequently would be amplified during library construction. It was shown that GC-rich regions of DNA are not effectively denatured during boiling due to high thermostability and thus, can lead to non-palindromic enrichment during GAPF.^35^ Because of these technical issues, 1 kb bins consistently enriched in both tumor and normal GAPF samples (75,765 bins, ~2.5% of the entire 3,209,513 bins) were removed in order to identify tumor-specific *de novo* DNA palindromes (Supp Table 2).

**Figure 3:**
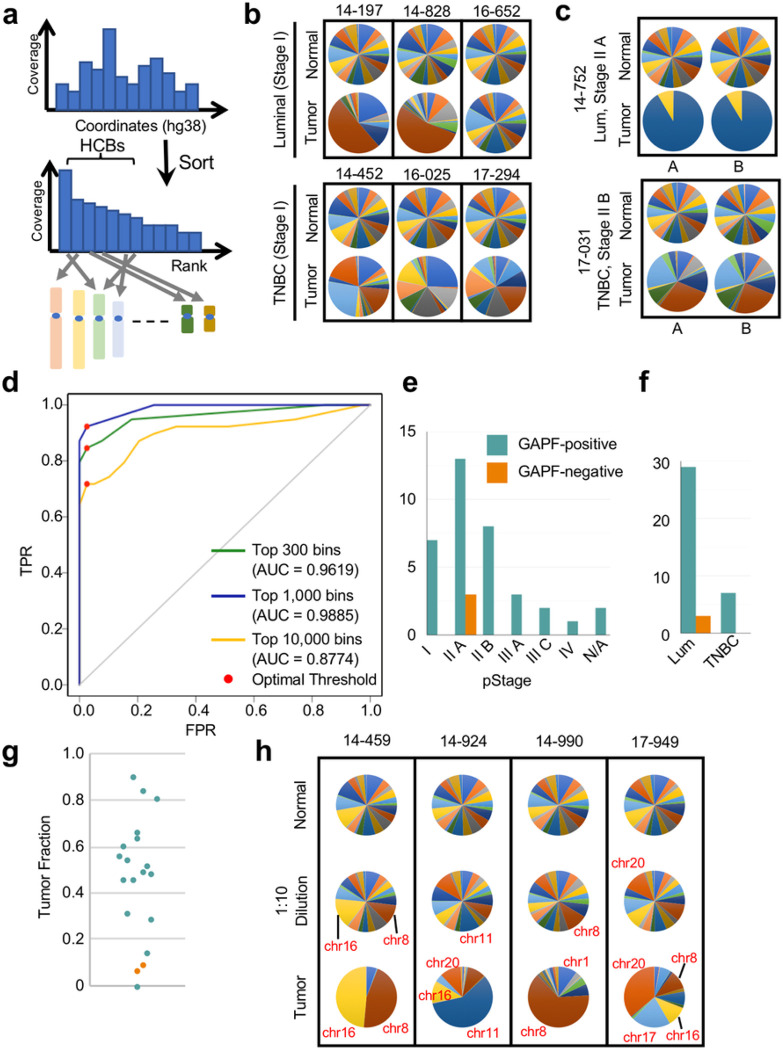
High coverage GAPF bins classifies tumor or normal DNA using a threshold model. **a** The analytical approach for GAPF-seq sorts 1 kb bins in descending order according to the normalized read coverage and identifies the chromosome of origin for the high coverage bins (HCBs) included in subsequent analyses. **b** GAPF profiles for breast tumors and matched normal buffy coat visualized in pie charts show clustering of the top 1,000 HCBs on chromosomes. **c** GAPF profiles using the top 1,000 HCBs are reproducible as visualized in pie charts for two breast tumor pairs performed in duplicate. **d** Receiver operating characteristic (ROC) curves generated by varying the threshold for the number of HCBs on a chromosome as the classification criterion for tumor or normal DNA. The top 300 (green), 1,000 (blue), or 10,000 (orange) HCBs were considered. The optimal threshold (red dot) is determined by Youden’s J statistic which maximizes sensitivity and specificity. **e** GAPF classification results for 39 breast tumors by stage using a chromosome threshold of 100 HCBs. **f**GAPF classification results for 39 breast tumors by subtype using a 100 HCB threshold. **g** Dot plot showing the tumor fraction (TF) by GAPF classification (blue = GAPF-positive, orange = GAPF-negative). n = 19. **h** GAPF profiles for normal DNA (top row), tumor DNA diluted 10x by normal DNA (middle row), and tumor DNA (bottom row).

The remaining 3,133,748 bins were ranked in descending order according to read depth coverage. The observation that HCBs were tumor-specific (Fig. 2C) led us to test the differential tumor/normal DNA calling using the chromosomal distribution of the top 1,000 HCBs (as GAPF profiles visualized in pie charts) (Fig. 3A).

It was observed that normal DNA GAPF profiles showed HCBs originating from almost every chromosome (Fig. 3B, Supp Fig. 4 and 5, Supp Table 3). In contrast, HCBs in tumor GAPF profiles appeared to originate primarily from one or a few chromosomes, possibly due to the clustering of *de novo* palindromic sequences in the breast tumor DNA. The even distribution of HCBs to all chromosomes in the normal GAPF profiles suggests randomness of residual dsDNA amplified before sequencing, as would be expected from a sample that does not contain any palindromes in single-copy regions of the genome analyzed. We also validated that GAPF profiles were highly reproducible (Fig. 3C, Supp Fig. 6). The apparent tumor-specific clustering of HCBs on chromosomes hinted at the intriguing possibility that a global threshold could be identified to classify samples as tumor or normal (threshold model) without prior knowledge of the precise locations of DNA palindromes.

To evaluate the optimal number of HCBs that can adequately differentiate tumor and normal DNA, receiver operating characteristic (ROC) curves were generated by taking the top 300, 1,000, or 10,000 HCBs and assessing the chromosomal distribution (Fig. 3C, Supp Table 3, 4, and 5). The classification criterion by which each sample was determined to be GAPF-positive or GAPF-negative was whether or not HCBs exceeded the global threshold on any one chromosome. For the top 1,000 bins, this global threshold varied from 70, at which point every sample was classified as GAPF-positive, to 915, at which point every sample was classified as GAPF-negative. The average area under the (ROC) curve (AUC) for the binary classifier using the top 1,000 HCBs was 0.9819 (s.d. ± 0.0249) with three-fold cross-validation and three different random assignment seeds. The optimal global threshold was then determined by Youden’s J statistic, which attempts to maximize the sensitivity and specificity of the binary classifier. Using the top 1,000 bins, the optimal threshold was determined to be 100 bins with a sensitivity of 94.02% (s.d. ± 2.96%) and a specificity of 99.15% (s.d. ± 1.48%). Using the top 300 or 10,000 HCBs instead yielded AUC values of 0.9628 (s.d. ± 0.0266) and 0.9050 (s.d. ± 0.0556), respectively, indicating that using the top 1,000 HCBs appeared to be the most effective for the binary classifier for this dataset.

Based on the top 1,000 HCBs and threshold model, there were 36 GAPF profiles with at least one chromosome above the 100 bin threshold (GAPF-positive) and 3 tumors misidentified as normal (GAPF-negative) (Fig. 3D). Notably, clustering of HCBs on a chromosome was apparent for all stage I tumors (TNBC or luminal), showing that GAPF-seq is able to detect early stage breast cancers with palindromes (Fig. 3B, Fig. 3D). The three GAPF-negative samples were all stage II A luminal breast tumors. Luminal tumors exhibit fewer SVs than TNBC,^40^ which might underlie GAPF-negative cases. Because SV often manifests as CNA, we quantified CNA for 31 breast tumors using shallow WGS and ichorCNA, a tool for estimating TF in plasma cell-free DNA (Fig. 3G). GAPF-negative tumors were low in CNA; however, several samples with low CNA were GAPF-positive (14–604, 16–094, and 16–263). To further examine tumors with very low CNA, we ran GAPF-seq with 10-fold *in vitro* dilutions of tumor DNA with paired normal DNA. All samples yielded the expected tumor DNA content after 10-fold dilution of tumor DNA. Based on the threshold (Fig. 3C), seven out of 8 samples were correctly called as tumor DNA suggesting that GAPF profiles can identify tumors with low DNA content (Fig. 3H, Supp Fig. 7, Supp Table 6).

### Subtype-specific Clustering of High Coverage Bins

**Figure 4:**
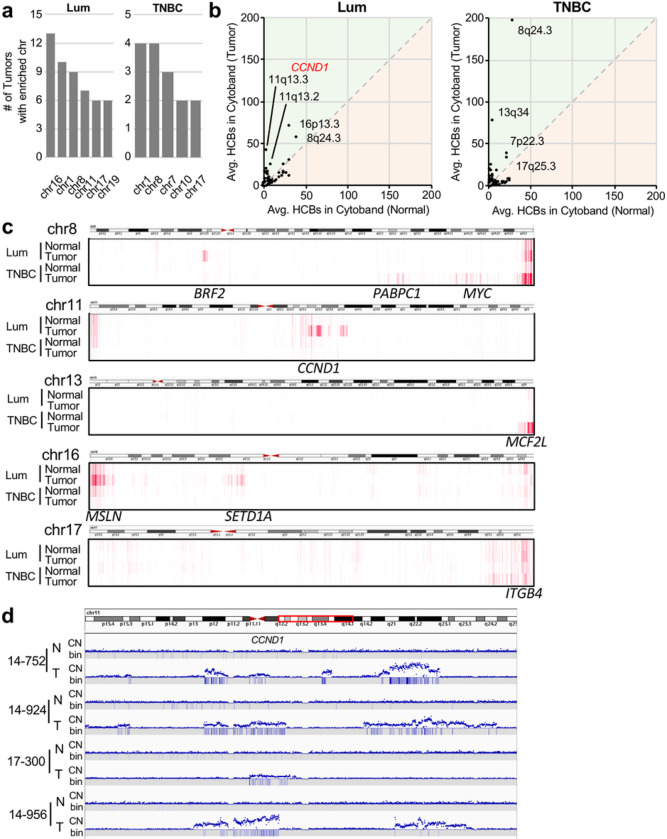
Clustering of high coverage bins is breast cancer subtype-specific. **a** The number of tumors (out of 39) with chromosomes enriched with high coverage bins (HCBs) above the threshold in Fig. 3D for luminal (Lum) and TNBC tumors. **b** The average number of HCBs in each cytoband in tumors is plotted against the number of HCBs in each cytoband in normal samples. Points above the diagonal indicate enrichment of the cytoband by GAPF. **c** A heatmap showing the location of HCBs on chr8, chr11, chr13, chr16, and chr17 for luminal and TNBC tumor and normal DNA. **d** CN and HCBs (bin) for four (4) luminal breast tumors near CCND1.

By looking at chromosomes frequently carrying top 1,000 HCBs above threshold, chr1 and chr8 appeared to be common between luminal and TNBC subtypes, whereas chr16 and chr11 were unique to luminal and chr7 was unique to TNBC (Fig. 4A). We also examined HCBs at the cytoband level where the number of GAPF top 1,000 HCBs in each cytoband from tumor GAPF profiles was compared to that of normal GAPF profiles (Fig. 4B). In TNBC on average, 197.6 of 1,000 HCBs were in 8q24.3, the telomeric end of 8q, in contrast to 28.0 HCBs in normal DNA. The 8q24.3 cytoband was also a hotspot for enrichment in luminal tumors and normal DNA (Fig. 4C). HCBs were often clustered in cytobands close to telomeres. In addition to 8q24.3, clustering was seen in the telomeric cytobands 13q34 (in TNBC) and 16p13.3 (in luminal) in a tumor subtype-specific manner (Fig. 4B, Fig. 4C). The fusions of critically short telomeres followed by BFB cycles^43^ may underlie the enrichment of palindromes and HCBs at the cytobands of chromosome ends. Notable exceptions include cytobands 8q24.21 (in TNBC) and 11q13.2–5 (in luminal), which harbor prevalent breast cancer oncogenes *MYC* and *CCND1*, respectively, and are frequently amplified. Indeed, the amplification of the *CCND1* locus was strongly associated with HCBs (Fig. 4D). Complex CNA patterns at 11q13 were common in luminal, with another amplicon telomeric to the *CCND1* amplicon associated with HCBs.

Aberrant read pairs spanning palindromic junctions are likely underrepresented,^38,39^ and deep coverage would be necessary to identify FF or RR read pairs (Fig. 1D). Nonetheless, we were able to find multiple RR read pairs at the copy number breakpoint of the amplicon at 11q13.2 with HCBs clustering nearby (Supp Fig. 8).

### Machine Learning Algorithms for Binary Classification

**Figure 5:**
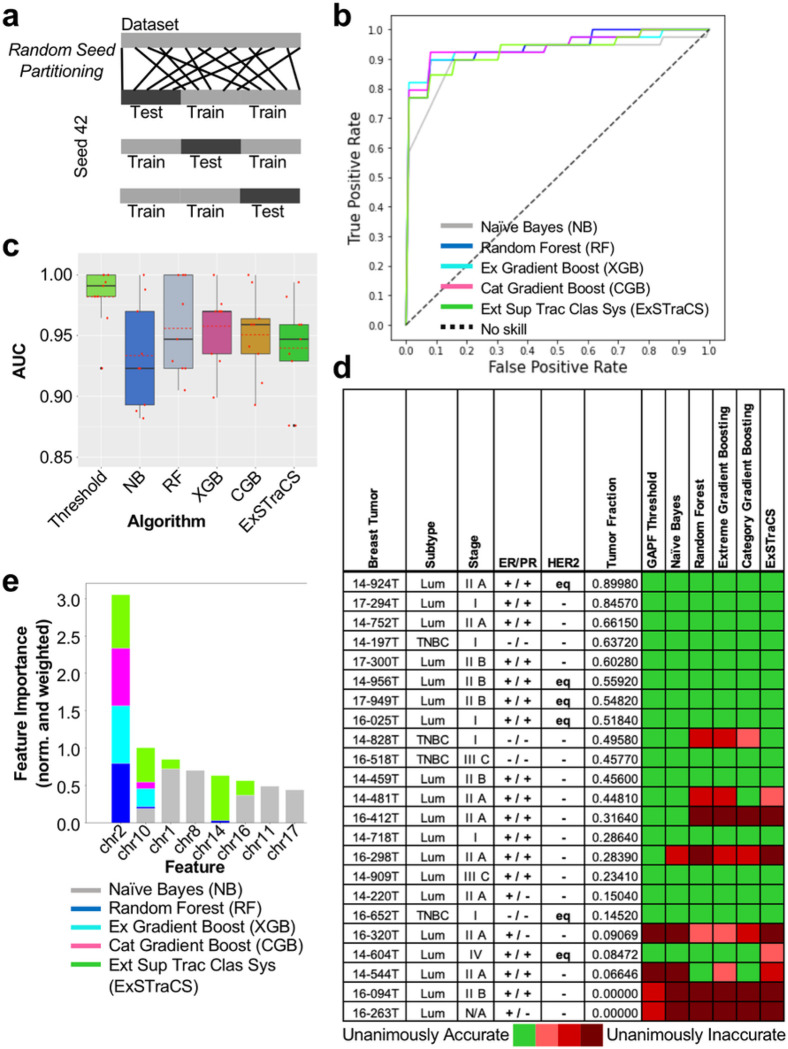
A machine learning pipeline with GAPF-seq accurately classifies tumor and normal DNA. **a** Random seed partitioning of the dataset assigns data points into groups for cross-validation (CV) testing of modeling. For three-fold CV, two groups are used to train the model while the remaining group is used to test the model. **b** ROC curves for machine learning (ML) models generated by STREAMLINE classifying tumor or normal DNA using the Naïve-Bayes (NB), random forest (RF), extreme gradient boosting (XGB), category gradient boosting (CGB), and extended supervised tracking and classifying system (ExSTraCS) algorithms with a random assignment seed of 42 and three-fold CV. **c** Box plots showing the AUC from each of 3 cross-validation testing sets for models generated using random assignment seeds of 22, 32, and 42. Red dot = AUC value, red dotted line = mean AUC. **d** Table of breast tumor subtype, stage, ER/PR/HER2 status, and tumor fraction (TF) with a heatmap showing the congruence of classification results from random assignment seeds and the threshold and machine learning (ML) models. **e** Composite normalized and weighted feature importance bar plots. Scores are normalized within each algorithm and weighted by the median AUC for that algorithm. Composite importance bar plots normalize feature importance scores within each algorithm and then weights these normalized scores by the median AUC for that algorithm to consider accurate models more than less accurate ones.

Aside from the threshold model for our dichotomous diagnostic test, a variety of statistical, probabilistic, and optimization techniques can be applied to genomic data using machine learning (ML) for binary classification. An automated end-to-end ML pipeline called STREAMLINE^44^ was used for the binary classification of the breast tumor and paired normal DNA (as in Fig. 3). The number of top 1,000 HCBs on each chromosome from the GAPF-seq data was input as a two-dimensional array and STREAMLINE attempted to identify the samples as tumor or normal. After basic data cleaning and three-fold cross-validation partitioning (Fig. 5A), ML models were trained using Naïve-Bayes (NB), random forest (RF), extreme gradient boosting (XGB), category gradient boosting (CGB), and extended supervised tracking and classifying system (ExSTraCS) algorithms. All models yielded very high AUC values (Fig. 5B). ML modeling was repeated using two additional random assignment seeds in order to minimize batch effects from partitioning, and the AUC results from all three seeds were averaged together (Fig. 5C). Average AUC values were greater than 0.93 for all models, with the best-performing ML model being created using XGB (Avg. AUC = 0.958). Each unique seed yields a predicted class for a sample, and so, a heatmap visualizes the congruence of true positive outcomes (Supp Table 7). Binary classification was mostly consistent between models, with a few exceptions (Fig. 5D). We found improvements over the threshold model in calling 14–544T, which was correctly called by RF and CGB models when tumors with low TF are often mis-classified as GAPF-negative as in Fig. 3G. In contrast, many non-linear prediction models incorrectly classified 14–828T, 16–412T, and 16–298T.

The automated ML pipeline STREAMLINE also estimated the relative importance of each chromosome toward accurately predicting tumor or normal DNA based on the GAPF profile (Fig. 5E). Feature importance was generated by randomly permutating one chromosome at a time in the testing data and evaluating its impact on the performance of each ML model.^45^ Interestingly, non-linear classification models RF, XGB, CGB, and ExSTraCS all deemed chr2 as most important. In particular, RF and CGB based their models almost exclusively on the GAPF HCB data from chr2, which stand in contrast to our threshold model. The top five chromosomes (chr2, chr10, chr14, chr15, chr18) identified as important for binary classification by RF, XGB, CGB, and ExSTraCS all demonstrated depletion in tumor GAPF profiles relative to normal DNA profiles. For example, normal samples had an average of 51.3 of the top 1,000 HCBs on chr2 whereas tumor tissue had only 24.3 (Supp Table 3). In contrast, for chromosomes frequently carrying HCBs above the threshold such as chr8 and chr11, the average numbers of HCBs were 51.3 and 64.8 in normal and 135.9 and 121.2 in tumor, respectively. Only the linear classifier algorithm Naïve-Bayes yielded high feature importance scores for chr1, chr8, and chr11, all of which were frequently above threshold in our global threshold model approach.

### Long cfDNA Fragments and Tumor Fraction

**Figure 6:**
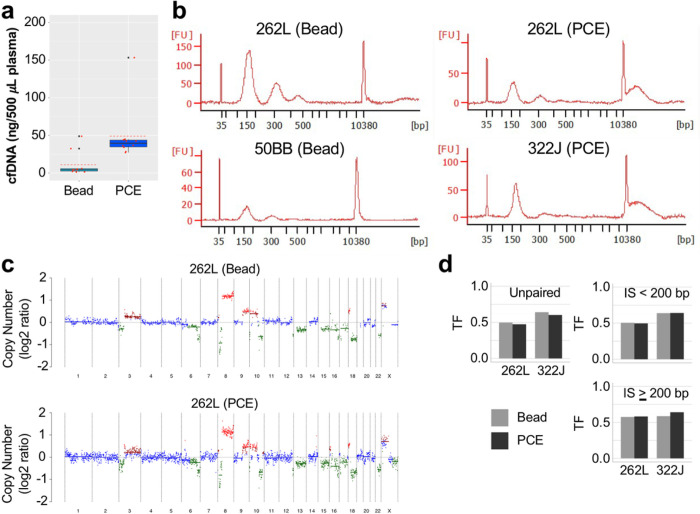
Long fragment cell-free DNA (cfDNA) extracted from plasma contains circulating-tumor DNA (ctDNA). **a** The amount of cell-free DNA (cfDNA) extracted from prostate cancer (PCa) patients using affinity-bead based extraction (bead) (n = 10) or phenol-chloroform extraction (PCE) (n = 10) normalized to 500 mL plasma. Red dotted line = mean. **b** Fragment analysis of cfDNA isolated from the blood of PCa patients using affinity-bead based extraction (bead) or phenol-chloroform extraction (PCE). **c** Copy number (CN) of cfDNA extracted using affinity beads (bead) or PCE as calculated by ichorCNA. **d**Tumor fraction (TF) for PCa cfDNA using unpaired sequencing reads (left) and paired sequencing reads with short (<200 bp) (top right) and long (>200 bp) (bottom right) insert sizes (IS).

With the ability to differentiate tumor DNA from paired normal DNA, we next tested GAPF-seq using plasma-borne cfDNA (cfGAPF-seq). For GAPF-seq, sufficiently long strands of dsDNA are needed so that both arms of DNA palindromes are physically tethered together in a continuous fragment to facilitate intrastrand annealing by snap-back. However, current mainstream methods for cfDNA extraction based on affinity-columns or -beads predominantly yield fragments bound to mono- or di-nucleosomes, which would be too short to capture palindromes. Whether very long ctDNA fragments exist in the plasma of cancer patients is under debate.^46^ To examine whether or not long cfDNA fragments exist and are as feasible for cancer detection as short cfDNA fragments, we isolated cfDNA using 0.5 mL plasma from 10 advanced prostate cancer (PCa) patients using either affinity-beads or traditional phenol-chloroform extraction (PCE) (Fig. 6A). Advanced PCa patient plasma should carry sufficient ctDNA for analyses whereas the majority of our breast tumor samples are Stage II or earlier and less likely to have high amounts of ctDNA in plasma. Additionally, the use of PCa cfDNA serves as an initial test for cfGAPF-seq as a pan-cancer approach. The bead-based cfDNA isolation kit extracted 11 ng of cfDNA per 500 mL of plasma on average with only two samples yielding more than 10 ng. In constrast, PCE yielded 49 ng on average with at least 27 ng from every sample. Because of the low amount of cfDNA extracted by the bead-based isolation kit, there often was not enough cfDNA for additional characterization experiments in addition to sequencing. Furthermore, whereas the bead-based cfDNA isolation kit yielded mono-, di-, and even some tri-nucleosomal cfDNA, extraction using PCE yielded a broad range of cfDNA fragment sizes in addition to the nucleosome-sized fragments (Fig. 6B). Notably, there were fragments longer than 10 kb, which would be amenable to snap-back. We then used fragmentation and NGS library construction for shallow WGS to evaluate TF using ichorCNA. Patterns in CNA appeared similar between affinity bead-extracted and PCE cfDNA (Fig. 6B and Fig. 6C). Slightly lower TF was observed in PCE cfDNA extracted from either 262L or 322J. To examine whether the decreased TF was due to lower TF in long fragments of the library, we evaluated TF for paired sequencing reads with short (<200 bp) and long (≥200 bp) insert sizes (Fig. 6D), assuming that read pairs with >200 bp insert sizes likely came from long DNA fragments after fragmentation rather than mononucleosomal fragments. TF was equally represented in both populations of size-selected reads indicating that long cfDNA fragments would be feasible for cancer detection.

### High Coverage Bins on chrX as a Determinant of Binary Classification

**Figure 7:**
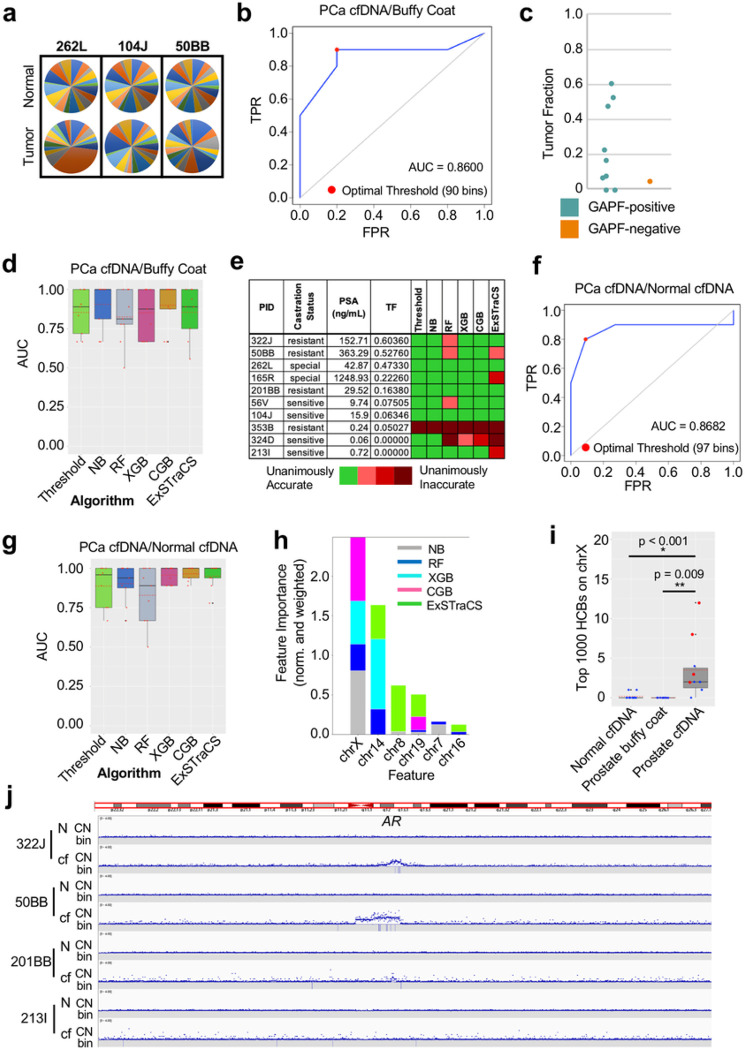
GAPF-seq accurately classifies PCa cfDNA and points to chrX as a key determinant factor. **a** GAPF profiles using the top 1,000 high coverage bins (HCBs) of prostate cancer (PCa) cfDNA and matched buffy coat DNA visualized in pie charts. **b**ROC curve generated by varying the threshold for the number of top 1,000 HCBs on a chromosome as the classification criterion for PCa cfDNA or normal buffy coat DNA. The optimal threshold (red dot) is determined by Youden’s J statistic. **c** Dot plot showing the tumor fraction (TF) by GAPF classification (blue = GAPF-positive, orange = GAPF-negative) (n = 10). **d** Box plots of AUC values for classifying PCa cfDNA and matched buffy coat DNA for machine learning (ML) classifiers using the Naïve-Bayes (NB), random forest (RF), extreme gradient boosting (XGB), category gradient boosting (CGB), and extended supervised tracking and classifying system (ExSTraCS) algorithms with three-fold CV and random assignment seeds of 22, 32, and 42. Red dot = AUC value, red dotted line = mean AUC. **e**Table of PCa castration status, PSA (ng/mL), and tumor fraction (TF) with a heatmap showing the congruence of classification results from random assignment seeds of 22, 32, and 42 and different classification models. **f** ROC curve for PCa cfDNA or healthy male cfDNA. The optimal threshold (red dot) is determined by Youden’s J statistic. **g** Box plots of PCa cfDNA and cfDNA from healthy male individuals showing the AUC from testing sets for models generated using random assignment seeds of 20, 32, and 42. Red dot = AUC value, red dotted line = mean AUC. **h**Composite normalized and weighted feature importance bar plots. Scores are normalized within each algorithm and weighted by the median AUC for that algorithm. **i** Box plots showing the number of top 1,000 HCBs on chrX for GAPF-seq data using healthy male cfDNA (n = 11), buffy coat DNA from PCa patients (n = 10), and cfDNA from PCa patients (n = 10). * p < 0.001, two-sided Wilcoxon rank-sum test. ** p = 0.009, Wilcoxon signed-rank test. Red dot = AUC value, red dotted line = mean AUC. **j** CN and HCBs (bin) for PCa cfDNA on chrX near AR.

GAPF profiles with the top 1,000 HCBs were generated from cfDNA along with matched leukocyte DNA from the buffy coat from 10 PCa patients (Fig. 7A, Supp Fig. 9, Supp Table 8). As with breast tumor and buffy coat, HCBs from the normal DNA were distributed to nearly every chromosome. In contrast, PCa cfDNA exhibited modest clustering of HCBs. An ROC curve with the top 1,000 HCBs showed that the AUC was 0.887 (s.d. ± 0.131) (Fig. 7B). The optimal global threshold determined by Youden’s J statistic was 90 bins, yielding a sensitivity of 87.04% (s.d. ± 15.65%) and a specificity of 79.63% (s.d. ± 21.29%). The TF for the cfDNA samples were grouped based on this output from GAPF profiles by our threshold model (Fig. 7C). Overall, the 5 samples with TF>0.1 were all called GAPF-positive. The lone false negative sample (353B) did not have the lowest estimated tumor fraction and two samples (324D and 213I) were correctly classified as GAPF-positive despite an estimated tumor fraction of zero. STREAMLINE was then used to generate binary classification models. The AUC values from each cross-validation set from 3 different models were averaged together and the best-performing model, on average, used the NB algorithm (Avg. AUC = 0.905) (Fig. 7D). A heatmap visualized the congruence of true positive outcomes against the castration status, prostate-specific antigen (PSA) level prior to treatment, and TF of PCa cfDNA (Fig. 7E, Supp Table 9). Increased PSA and TF correlated with more accurate classification by GAPF-seq and castration resistance.

Since plasma cfDNA and DNA extracted from the buffy coat have different pre-analytical steps, the binary classification results could ostensibly result from differences in sample processing. cfGAPF-seq was performed on 11 plasma samples from males without any known diseases (Supp Fig. 10, Supp Table 10). [mention AUC] (Fig. 7F). These healthy “normal” plasma samples were classified along with the 10 plasma samples from PCa patients using the threshold model approach and ML modeling. The average AUC values from three different models for each algorithm were averaged together, and the best-performing model used the CGB algorithm (Avg. AUC = 0.965) (Fig. 7G).

Models using NB, RF, XGB, and CGB all considered chrX as the first or second most important feature for binary classification (Fig. 7H). Because none of the samples had a seemingly significant number of top 1,000 bins on chrX, our threshold-based calling was insensitive to chrX. Therefore, we independently evaluated chrX HCBs in calling tumor cfDNA (Fig. 7I). While cfDNA from healthy individuals or buffy coat DNA from PCa patients contained zero or one top 1,000 HCBs, quite a few of these bins were mapped to chrX in cfDNA GAPF profiles from PCa patients. Indeed, HCBs on chrX are statistically different between PCa cfDNA and either matched normal DNA (p < 0.001, Wilcoxon signed-rank test) or healthy individuals’ cfDNA (p = 0.009, two-sided Wilcoxon rank-sum test). Because the androgen receptor gene (AR) is located on chrX and causes therapy-resistance in advanced PCa^47–49^, we examined AR amplification (AR-amp) by shallow WGS. AR-amp was notable in four patients with therapy-resistant tumors and very high PSA, with dozens of top 1,000 bins within the amplified regions (Fig. 7J). In other patients, AR-amp was not evident, and HCBs were fewer and scattered throughout chrX. These PCa were therapy-sensitive at the time of the blood draw but would eventually progress to resistance and life-threatening disease. HCBs on chrX, as assessed by cfGAPF-seq could be an initial sign of chrX-wide rearrangement that would eventually leads to AR-amp, which could be consistent with DNA palindrome formation as an initial step of gene amplification.

## Discussion

In this study, we tested the feasibility of our genome-wide assay targeting DNA palindromes (GAPF-seq) for liquid biopsy-based cancer detection. Because DNA palindromes are mechanistic components of BFB cycles, which are crucial drivers of genome instability and genomic amplification, targeting DNA palindromes for cancer detection seemed a rational strategy to test. GAPF is designed to enrich DNA palindromes and eliminate non-palindromic DNA. Therefore, GAPF-seq could detect cancer-specific DNA with high sensitivity in plasma cfDNA with low TF. The idea was tested in this study using samples with *in vitro* serial dilution of tumor DNA with normal DNA, and cfDNA with various TFs.

GAPF-seq would be uniquely positioned for liquid biopsy targeting SVs if developed as a cancer detection test. Fold-back inversions can be identified successfully by WGS from tumor DNA.^26,50^ However, WGS requires decent coverage (>30x). In cfDNA, DNA representing fold-back inversions are diluted, the detection of which would require ultra-deep coverage. Ultra-deep WGS would be cost-inhibitory and requires significant time and effort for analyses. Targeted sequencing for gene mutation detection in cfDNA would have a higher sensitivity for cancer detection, as exemplified by detecting tumor-informed mutations.^51,52^ However, amplified oncogenes lack mutations in most instances. Gene amplification not only occurs in treatment-naïve primary tumors for activating oncogenes but also occurs later during disease progression to confer therapy resistance, which primary tumors would not inform. Early detection of resistance would allow physicians to consider treatment options early when resistant tumors are small. Thus, frequent monitoring by liquid biopsy and timely detection of gene amplification is important. GAPF-seq with simple cfDNA processing, low coverage NGS (~7.5x coverage), and streamlined data analysis seems practical for frequent monitoring.

The proof of concept was tested in this study for AR-amp in lethal PCa. AR-amp is a major genomic change associated with advanced PCa.^49^ AR signaling is a major driver of PCa progression. In recent years, contemporary AR signaling inhibitors (e.g. enzalutamide, apalutamide, abiraterone) have significantly improved the overall survival of patients.^53,54^ Despite these advances, resistance eventually develops. The underlying CRPC eventually metastasizes leading to death. The only indication of resistance is a rising PSA which is typically only verified later. Given mounting data pointing toward the benefits of early initiation of chemotherapy, a test indicating resistance to AR suppression early would provide an opportunity to pivot the therapeutic approach that could potentially result in improved survival.^47^ We found that genomic regions enriched by GAPF on chrX were almost exclusively seen in PCa plasma cfDNA (Fig. 7I): all four cases with anti-AR therapy resistance and five out of six therapy-sensitive cases. Resistance cases were associated with high TF in cfDNA and AR-amp (Fig. 7J). These results may suggest that palindrome formation and BFB cycles underlie AR-amp. The other six cases did not show AR-amp; however, HCBs scattered throughout chrX. Such bins may be early signs of BFB cycles and chromosome-wide rearrangements that eventually lead to AR-amp. If that is the case, these patients will eventually develop AR-amp. If not, HCBs in chrX could be a simple indicator of cancer DNA. Systematic studies with longitudinally collected early and late-stage samples would distinguish these two possibilities.

We employed chromosomal distributions of HCBs (GAPF profiles) as outputs from GAPF-seq and tested their ability to distinguish breast tumor DNA samples from normal DNA. The output worked well in most cases, but several tumors (luminal) DNA and at least one cfDNA case are inconsistently classified. We found that all of these cases had low CNA measured by ichorCNA. It is possible that tumor-derived DNA was very rare in these samples. This is most likely the case for PCa cfDNA, because disease burden, estimated by PSA, was low for inconsistently classified or GPAF-negative cases. It is also possible that GAPF-seq profiles are not a good measure of tumor DNA when chromosomal rearrangements are rare. In this regard, all three GAPF-negative cases were from luminal tumors, which are known to be driven by hormone-dependent signaling.^55^ Understanding the power of GAPF-seq to differentiate tumor DNA from normal DNA for each tumor tissue of origin would thus be necessary.

In many cases, the relatively straightforward threshold approach yielded better binary classification results with respect to AUC than the ML models in many cases. In contrast with the ML models, which only receive HCBs as input data, the simple threshold models have the advantage of incorporating domain knowledge; i.e. (1) the understanding that a burden count above some threshold may be informative and (2) there is an informative higher-level feature abstraction, i.e. one or more chromosomes have a burden above the target threshold. In order for a machine learning algorithm to train a model similar to the threshold models, it would need to first learn the abstract concept which decides “IF” any of the HCB features in the dataset have a value over a target threshold. The challenge of this abstraction is reflected in long standing computer science benchmark problems such as the “majority-on” or “even-parity” problems wehre simultaneousl knowledge of multiple features values is required to solve the problem (i.e., the majority of features in a set above some threshold, or the sum of feature values in a set is even, respectively).^56,57^ This ML challenge can be addressed in future work via feature engineering; applying domain knowledge to construct new features that capture information across all chromosomes (e.g. construct a binary feature identifying if any chromosome in the dataset has an HCB above a given threshold). This would be expected to improve ML binary classification. Beyond feature engineering, additional opportunities to improve the predictive performance of GAPF-seq with ML include (1) utilizing more sophisticated ML algorithms, (2) conducting a more extensive optimization of algorithm hyperparameters, and (3) combining base models trained by different ML algorithms into a predictive ensemble.

Nevertheless, utilizing chrX-based tumor/normal differentiation for PCa cfDNA/normal DNA was guided by insight from ML (Fig. 7I). Because HCBs were few in chrX, none of the cfDNA samples reached the threshold. However, the estimation of feature importance revealed that ML algorithms strongly relied on HCBs from chrX for binary classification (Fig. 7H). Thus, ML pointed to a feature that humans (i.e the authors) did not take into consideration. Feature importance is the most popular technique to explain why the ML models behave the way they do, the importance of which is pronounced in the medical field.^58^ The importance of DNA palindromes in chrX is easily interpretable, given that AR-amp is a primary driver of resistance to therapy.^47–49^ These results suggest that GAPF-seq (via the threshold model) has limitations, and we have not exhausted the potential of GAPF-seq for calling tumor DNA. Increasing the number of samples in future studies will allow us to improve analysis pipeline. Increasing the resolution of HCB positioning in 1,293 cytobands genome-wide may provide a better assessment. For example, cfDNA fragmentation patterns were examined in 504 windows of 5 Mb genome-wide.^23,24^ DNA methylation profiling used over 1 million CpG sites in the genome, the methylation patterns of which best predict the tissue of origin and differentiate tumor DNA from normal DNA.^59^ The high demand for computational power can be managed by employing ML. Feature importance would be able to point to hotspots of DNA palindromes at the cytogenetic level.

### Limitations of the study

- While GAPF-seq was able to efficiently differentiate tumor DNA from normal DNA in breast and prostate tumors, it is unknown whether GAPF-seq would do so for other tumor types.
- HCBs in chrX seems definitive for calling tumor DNA. However, studies with increased sample size are necessary to make a strong case.

## Methods

### Research Model:

We used a colorectal cancer cell line Colo320DM containing a known palindromic junction at the CTSK gene and IMR90 normal fibroblasts. Breast samples were obtained from the BioBank at Cedars-Sinai Medical Center, which has stored matched frozen tumor/buffy coat/plasma samples. The project was approved by the Institutional Review Board (Pro0005127, DNA palindrome detection by liquid biopsy from breast cancer patients; 00042197 Translational Oncology Program Blood Specimen Repository). Human normal plasma from single donors was obtained from Innovative Research (IPLASK2E2ML).

### DNA extraction:

High Molecular Weight DNA from cell lines and tissues were extracted as described previously.^38^ Briefly, samples were incubated in 400 mL of lysis buffer and 20 mL of Proteinase K (20 mg/mL) at 37 °C for 16 hr, followed by phenol/chloroform extraction and ethanol precipitation. Gene Jet Whole Blood DNA extraction kit was used to extract DNA from buffy coat. Cell-free DNA from plasma was extracted using a phenol-chloroform protocol. Frozen aliquots of 500 mL of plasma were thawed and mixed with 2.5 mL of cell lysis buffer (100 mM NaCl, 10 mM Tris-HCl, 25 mM EDTA, and 0.5% SDS) and 200 mL proteinase K (20 mg/mL) and incubated at 37°C for at least 16 hr. A 1:1 phenol-chloroform mixture was added and mixed. The mixture was centrifuged at 10,000 g for 15 min and the aqueous portion was kept for a second phenol-chloroform wash. A 1:1 phenol-chloroform mixture was then added and mixed followed by centrifugation at 10,000 g for 15 min. To the aqueous portion, 0.05 mg of glycogen and sodium acetate to a final concentration of 0.3 M were added. Then, 2:1 absolute ethanol was added and incubated at −20°C overnight. The mixture was centrifuged at 14,000 g for 30 min at 4°C and the pellet was washed with 70% ethanol and resuspended in low TE buffer. cfDNA concentration was measured using the dsDNA HS Assay Kit (Life Technologies, Q32851) and a Qubit 3.0 fluorometer. DNA fragment analysis was performed using an Agilent 2100 Bioanalyzer system.

### Genome-wide Analysis of Palindrome Formation:

We used a modified protocol of the Genome-wide Analysis of Palindrome Formation (GAPF)^35^ for small amounts of DNA. Briefly, either 100 ng or 30 ng of input genomic DNA was digested with either KpnI or SbfI, both of which are relatively infrequent cutting enzymes, at 37°C for 16 hr. The restriction enzymes were heat-inactivated at 65°C for 20 min and the reaction products were mixed. The mixture was boiled for 7 min in 100 mM NaCl and 50% formamide and immediately quenched in ice water for 5 min. DNA was digested with 100 U/mg of S1 nuclease at 37°C for 1 hr and processed with the ChargeSwitch PCR Clean-up Kit or Monarch PCR and DNA Cleanup Kit.

Purified DNA was fragmented using the Covaris M220 and the libraries were constructed using the NEBNext Ultra II DNA Library Prep Kit for next generation sequencing. Alternatively, GAPF libraries were constructed with the NEBNext Ultra II FS DNA Library Prep Kit, which uses fragmentase. Whole genome sequencing libraries were constructed using 40 ng of genomic DNA and the NEBNext Ultra II FS DNA Library Prep Kit.

### Whole Genome Sequencing:

All samples were sequenced on the Illumina HiSeq System (Novogene Co., Ltd.) to generate 150 bp paired-end sequencing reads at approximately 7.5x coverage. The reads were trimmed to remove adapters using Trim Galore (v0.6.1) and Cut Adapt (v2.3) (*trim galore options: --length 55*) and aligned to the UCSC hg38 human reference genome (hg38.fa.gz, 2014–01-15 21:14) using the DNA alignment software Bowtie 2 (v2.3.5) (*options: -x -U -S or -x −1 −2 -S*). Reads were converted to binary format (*samtools view options: -bS*), reordered (*samtools sort options: -o*), and sorted (*sort options: -k1,1 -k2,2n - o*) using Samtools (v1.9) and counted in genome-wide 1 kb non-overlapping bins using Bedtools (v2.28.0) (*bedtools coverage options: -sorted -counts -a -b -g*). Coverage was visualized using the Integrative Genomics Viewer (IGV) (v2.5.0). Read counts were normalized by a per-million scaling factor to adjust for the sequencing depth.

### GAPF-seq Quality Control:

To determine the quality of palindrome amplification by GAPF-seq, we validated the sequencing data using known inverted repeats on chromosomes 1, 9, 17, 19, and X (Supp Table 11). These short, inverted repeats are infrequently cut by the restriction enzymes and readily amplified by GAPF. Reads were counted at the inverted repeats at the X chromosome genes DMRTC1, SSX2, SSX4, CXorf49, CXorf51A, PNMA6A, and SPANXA2 as well as the inverted repeats at HIST2H3C on chr1, FAM225A on chr9, SNORD3A on chr17, and 19p13.2. The normalized read counts for each of the regions were averaged to determine an amplification score. Because these regions are repeated within the reference genome, filtering based on mapping quality score that only returns reads that uniquely map to the reference genome should deplete the normalized read counts (*samtools view options: -b -q 40*). Thus, filtered values for each region were averaged to determine a depletion score. GAPF-seq passes quality control with an amplification score above 1.0 and a depletion score less than 0.075 demonstrating adequate enrichment of DNA palindromes and elimination of non-palindromic or repeated sequences.

### GAPF Background Bins:

Due to their repetitive structure and high GC content, certain regions of the genome such as Alus, LINE elements, short tandem repeats, and segmental duplications can result in false positives or obscure the identities of DNA palindromes. To address this, we first applied a filter based on mapping quality score (MAPQ > 40) that returns the reads that uniquely map to the reference genome (hg38). Then, we removed regions that were commonly enriched in both tumor and normal samples. Out of the original 3,209,513 non-overlapping 1 kb bins, this curation resulted in 3,133,748 bins for further GAPF analysis. (Supp Table 2).

### Cytoband Analysis:

Coordinates for cytogenetic bands in hg38 were downloaded from the UCSC genome browser (cytoBand.txt.gz, 2022–10-28 21:15). After removing the GAPF background bins (Supp Table 2), reads were counted in cytobands in order to calculate the coverage for each cytoband. There were 1,293 cytobands downloaded from the UCSC genome browser. Unlocalized sequences were removed from the analysis leaving a total of 863 cytobands for subsequent analyses. For figures containing 1-kb HCBs in cytobands, the number of top 1,000 high coverage GAPF bins corresponding to each cytoband were counted.

### Tumor Fraction:

DNA copy number (CN) and tumor fraction (TF) were calculated from shallow whole genome sequencing data using the hidden Markov model in the software package ichorCNA (v0.3.2).^60^ (*read counter options: --window 1000000 --quality 20 --chromosome “chr1, chr2, chr3, chr4, chr5, chr6, chr7, chr8, chr9, chr10, chr11, chr12, chr13, chr14, chr15, chr16, chr17, chr18, chr19, chr20, chr21, chr22, chrX, chrY”) (runIchorCNA options: --ploidy “c(2,3)” --normal “c(0.5,0.6,0.7,0.8,0.9)” --maxCN 3 --gcWig gc_hg38_1000kb.wig --mapWig map_hg38_1000kb.wig --centromere GRCh38.GCA_000001405.2_centromere_acen.txt --normalPanel HD_ULP_PoN_hg38_1Mb_median_normAutosome_median.rds --includeHOMD False --chrs “c(1:22, \”X\”)” --chrTrain “c(1:22)” --estimateNormal True --estimatePloidy True --estimateScPrevalence False --scStates “c()” ---txnE 0.9999 --txnStrength 10000)*

### GAPF Binary Classifier (Threshold Model):

The normalized read coverage in 1 kb non-overlapping bins following the mapping quality score filter (MAPQ score >= 40) were sorted in descending order (*sort options: -nr -k 4 -k 2n*) and the top 1,000 bins according to coverage were considered for subsequent analyses (*head options:* −*1000*). The number of these high coverage bins (HCBs) corresponding to each chromosome were counted (totaling 1,000). A binary classifier was established designating samples as GAPF-positive or GAPF-negative according to the number of HCBs on a chromosome against a threshold. Using the 39 breast tumor and normal samples obtained by the BioBank, the sensitivity and specificity were calculated at various thresholds for HCBs on a chromosome. A receiver operating characteristic curve (ROC) was generated and the area under the curve (AUC) was calculated using the trapezoidal rule. The optimal threshold was determined using Youden’s J statistic. For the 39 breast tumor and buffy coat pairs, the optimal threshold was determined to be 100 bins on any chromosome for the breast sample to be classified as GAPF-positive.

### Automated Machine Learning Pipeline (STREAMLINE):

Machine learning binary classification modeling and evaluation was conducted using version 0.2.5 of the Simple Transparent End-To-End Automated Machine Learning Pipeline (STREAMLINE)^44^ applying mostly default pipeline run parameters (exceptions identified below). STREAMLINE is a recently developed AutoML tool that facilitates ML modeling while enforcing rigorous modeling algorithm comparisons, analysis transparency, reproducibility, and sensitivity to complex associations in data. In the first year of its release it has been successfully applied to various biomedical ML modeling and data mining tasks.^61,62^ Input data were the number of top 1,000 HCBs on chromosomes 1–22 and X (23 features) unless otherwise stated. Three-fold cross-validation (CV) was used in the predictive modeling using the following classification algorithms: Naïve-Bayes (NB), Random Forest (RF), Extreme Gradient Boosting (XGB),^63^ Category Gradient Boosting (CGB),^64^ or Extended Supervised Tracking and Classifying System (ExSTraCS).^65^ Standardized within STREAMLINE, all algorithms with hyperparameters (excluding NB) undergo an Optuna-driven hyperparameter sweep^66^ with 200 target trials and a timeout of 15 min across a broad range of hyperparameter options. In addition to evaluating all models across 16 classification metrics, STREAMLINE also calculates model feature importance estimates uniformly for each algorithm and model using permutation feature importance estimation. STREAMLINE model training was repeated for each analysis using random seeds of 22, 32, or 42 unless otherwise stated.
